# The evidence for Shiatsu: a systematic review of Shiatsu and acupressure

**DOI:** 10.1186/1472-6882-11-88

**Published:** 2011-10-07

**Authors:** Nicola Robinson, Ava Lorenc, Xing Liao

**Affiliations:** 1 Allied Health Sciences Department, Faculty of Health and Social Care, London South Bank University, 103 Borough Road, London SE1 0AA, UK; 2 Institute of Basic Research in Clinical Medicine, China Academy of Chinese Medicial Sciences 16 Dongzhimeng, Nanxiaojie, Beijing, 100700, China

## Abstract

**Background:**

Shiatsu, similar to acupressure, uses finger pressure, manipulations and stretches, along Traditional Chinese Medicine meridians. Shiatsu is popular in Europe, but lacks reviews on its evidence-base.

**Methods:**

Acupressure and Shiatsu clinical trials were identified using the MeSH term 'acupressure' in: EBM reviews; AMED; BNI; CINAHL; EMBASE; MEDLINE; PsycARTICLES; Science Direct; Blackwell Synergy; Ingenta Select; Wiley Interscience; Index to Theses and ZETOC. References of articles were checked. Inclusion criteria were Shiatsu or acupressure administered manually/bodily, published after January 1990. Two reviewers performed independent study selection and evaluation of study design and reporting, using standardised checklists (CONSORT, TREND, CASP and STRICTA).

**Results:**

Searches identified 1714 publications. Final inclusions were 9 Shiatsu and 71 acupressure studies. A quarter were graded A (highest quality).  Shiatsu studies comprised 1 RCT, three controlled non-randomised, one within-subjects, one observational and 3 uncontrolled studies investigating mental and physical health issues. Evidence was of insufficient quantity and quality. Acupressure studies included 2 meta-analyses, 6 systematic reviews and 39 RCTs. Strongest evidence was for pain (particularly dysmenorrhoea, lower back and labour), post-operative nausea and vomiting. Additionally quality evidence found improvements in sleep in institutionalised elderly.  Variable/poor quality evidence existed for renal disease symptoms, dementia, stress, anxiety and respiratory conditions. Appraisal tools may be inappropriate for some study designs. Potential biases included focus on UK/USA databases, limited grey literature, and exclusion of qualitative and pre-1989 studies.

**Conclusions:**

Evidence is improving in quantity, quality and reporting, but more research is needed, particularly for Shiatsu, where evidence is poor.  Acupressure may be beneficial for pain, nausea and vomiting and sleep.

## Background

Shiatsu is a form of complementary and alternative medicine (CAM) which primarily developed in Japan [1]. Both Shiatsu and acupressure have roots in Chinese medicine and embrace the philosophy of Yin and Yang, the energy meridians, the five elements and the concept of Ki, or energy. This concept of affecting the balance of energy through acupoints on the meridians is similar to acupuncture where needles or heat is applied to acupoints [2]. 'Shiatsu' literally means "finger pressure", but uses gentle manipulations, stretches and pressure using fingers, thumbs, elbows, knees and feet. Shiatsu incorporates acupressure, which is similar but applies pressure for longer on specific pressure points on meridians, following Traditional Chinese Medicine (TCM) theory. Shiatsu tends to cover the whole body[3]. Shiatsu diagnosis is primarily through touch, rather than TCM which primarily uses the pulse diagnosis and inspection of the tongue. Shiatsu practitioners are trained in the anatomical location, functions and uses over 150 pressure points on the body. Evidence for the efficacy of acupressure may therefore potentially support claims about the efficacy of Shiatsu [4].

Shiatsu is practiced in many European countries but varies in styles, philosophical approaches and theoretical bases. The approaches most commonly found in Britain are Zen Shiatsu, Macrobiotic Shiatsu, Healing Shiatsu, Tao Shiatsu, Seiki, Namikoshi Shiatsu and Hara Shiatsu) [3,5].

Shiatsu aims to balance, restore and maintain the body's energy balance and prevent the build up of stress in the UK. The most common conditions presenting for treatment are musculo-skeletal and psychological problems[6]. Health problems which may be amenable to treatment by Shiatsu include: headaches, migraine, stiff necks and shoulders, backaches, coughs, colds, menstrual problems, respiratory illnesses including asthma and bronchitis, sinus trouble and catarrh, insomnia, tension, anxiety and depression, fatigue and weakness, digestive disorders and bowel trouble, circulatory problems, rheumatic and arthritic complaints, sciatica and conditions following sprains and injuries [3]. Shiatsu is, however, a holistic therapy and often also impacts a patient's well-being, lifestyle, diet, body/mind awareness [7]. Shiatsu is commonly used by older (median age of 50 in the UK) females [7].

This review aimed to identify the evidence base informing the practice of Shiatsu. Due to the lack of Shiatsu specific literature and overlap in practice and theory, acupressure studies were also included. Although there are a number of systematic reviews for acupressure, they were mostly confined to a single (Western) condition such as nausea and vomiting [8] or dysmenorrhoea [9].

### Objectives

To systematically review all papers using Shiatsu or acupressure for any health condition for any population, using either a systematic review/meta-analysis, RCT, quasi-experimental, or uncontrolled design.

## Methods

### Eligibility criteria

Inclusion criteria were:

• Shiatsu or acupressure administered manually/bodily

• Meta-analysis, systematic review or clinical trial

• Published after January 1990

Exclusions were:

• Guidelines for treatment, reports of possible adverse events, surveys, case reports/series, non systematic reviews, qualitative studies, conference abstracts/posters

• Newspaper articles, book reviews, 'popular' health publications, general comments or letters.

• Papers included in systematic reviews included in this review

• Papers in a language other than English

• Use of plasters, devices, or wristbands

• Acupressure on auricular or Korean points/meridians

### Information sources

Databases searched were: EBM reviews (includes all Cochrane Library resources); Allied and Complementary Medicine (AMED);British Nursing Index (BNI);Cumulative Index to Nursing & Allied Health Literature (CINAHL); EMBASE; MEDLINE; PsycINFO/PsycARTICLES; Science Direct; Blackwell Synergy; Ingenta Select; Wiley Interscience; Index to Theses and ZETOC (British Library electronic table of contents). In addition the references of retrieved articles were checked to identify any further studies.

### Search

The MeSH term tree 'acupressure' was used which incorporates Shiatsu. For databases not using MeSH terms, 'shiatsu' or 'acupressure' were used.

### Study selection

Study selection was independently performed by two reviewers using the inclusion/exclusion criteria given above, followed by discussion and consensus within the research team. The first stage of selection used the abstracts, the second stage the full text of the papers.

### Data collection process

For each study the following data was extracted independently by two reviewers using a standardised extraction form. Any disagreements were moderated by a third reviewer.

• Authors

• Date

• Study design (meta analysis, systematic review, randomized controlled trial, case control trial or uncontrolled study)

• Health condition

• Setting

• Sample

• Intervention

• Outcome measures

• Results

• Conclusion

### Quality assessment

The contribution made to the evidence base by each study, based on the study design, rigour of methods and reporting, was evaluated independently by two reviewers, with an independent adjudicator. Studies were evaluated on the following quality indicators to determine its contribution to the evidence base:

• The rigour of the study conducted was determined using a critical appraisal checklist [10]

• Adapted STRICTA score for quality of reporting of the intervention (acupressure only, not Shiatsu) for each study [11] (reported as a score out of 16 relevant items - item 2 g on STRICTA, needle type was not relevant)

• Quality of reporting, assessed using established checklists: CONSORT guidelines for RCTs[12]; CASP guidelines for systematic reviews [13]; and TREND statement for non-randomised studies [14].

• Study design (according to the hierarchy meta-analysis > systematic review > RCT > controlled trial > uncontrolled trial), as discussed in the NICE guidelines manual, section 6 [15].

Studies were graded A (good), B (fair/moderate) or C (poor) depending on these indicators. Results of this evaluation are given for each study in Additional file 1.

### Synthesis of results

Studies were grouped into either Shiatsu or acupressure and within these categories according to health condition treated. For each health condition evidence was categorised according to criteria from Waddell [16].

Category 1: Generally consistent finding in a range of evidence from well-designed experimental studies

Category 2: Either based on a single acceptable study, or a weak or inconsistent finding in some multiple acceptable studies.

Category 3: Limited scientific evidence, which does not meet all the criteria of acceptable studies, or an absence of directly applicable studies of good quality. This includes published and unpublished expert opinion.

This review has been reported according to the principles in the PRISMA statement [17] and acupoints are reported using the WHO system [18]

## Results

### Study selection

**Figure 1:**
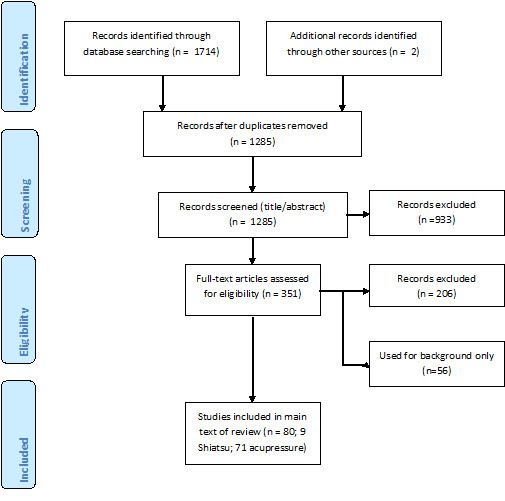
**Flowchart of study selection**.

After carrying out the database searches, a total of 1714 publications were identified (Figure 1). After duplicate items, newspaper articles and commentaries were removed 1285 items remained. From screening the abstracts 933 articles were excluded. Two reviewers screened the full texts of the remaining 351 articles using exclusion criteria and quality assessment and excluded 206. Of those remaining, 56 were used for background information only, leaving 89 studies. A further 9 were excluded as they were already included in systematic reviews included in this review. The total included studies were 9 Shiatsu and 71 acupressure publications.

Details of included studies are presented in Additional file 1, grouped by health condition. Just under one third (27.5%) were graded A (highest quality), 42.5% graded B and 26.3% C (lowest quality) (3 studies were ungraded); this grading refers to the contribution the study made to the evidence, which took into account study design, rigour and reporting.

### Shiatsu

Only 9 Shiatsu studies were of sufficient quality to be included in the review. These comprised 1 randomised controlled trial (RCT), three controlled non-randomised, one within-subjects trial, one observational study and 3 uncontrolled studies. These studies investigated quite separate health issues, did not use comparable methodology and data could not be pooled due to their heterogeneity. Subjects were chronic stress, schizophrenia, promoting well-being and critical health literacy, angina, low back and shoulder pain, fibromyalgia, chemotherapy side effects/anxiety and inducing labour. They are grouped by methodology and discussed below.

One RCT was identified (integrated care, which included Shiatsu), for back and neck pain [19]. No significant effects, compared to standard care were identified. The study used a fairly large sample (n = 80) but was underpowered to detect any statistically significant effects.

Three studies compared two or more treatments with non-random group allocation, rather by preference [20], participants in another study [21] or staff on duty [22]. Lucini et al [20] evaluated Shiatsu for chronic stress; 70 volunteer patients chose either active (relaxation and breathing training), passive (Shiatsu) or sham treatment (stress management information). Small sample, limited the validity of results. Although the design accounted for patient preference, results were confounded by more stressed patients choosing sham. Ingram [22] compared Shiatsu to no intervention for post-term pregnancy in 142 women. The Shiatsu group was significantly more likely to labour spontaneously than the control (p = 0.038) and had a longer labour (p = 0.03), but groups were allocated according to which midwife was on duty (although groups were homogenous for maternal age, parity and delivery details). Ballegaard et al [21] conducted a study of cost-effectiveness and efficacy of Shiatsu for angina pectoris. Sixty-nine consecutive patients were treated and compared with those from a separate trial of two invasive treatments for angina[23]. Incidence of death/myocardial infarction (MI) was 7% in this sample, compared to 21% and 15% in the comparison group with no significant difference in pain relief. Additionally a cost-saving of $12000 per patient was estimated. The groups were from different countries (USA and Denmark), additionally 56% of the participants would have been excluded from the one of the comparison groups. It also used a convenience and unpowered sample and no blinding.

One study used a within-subjects repeated measures design, comparing Watsu (water Shiatsu) with Aix massage for fibromyalgia syndrome [24]. A significant improvement was seen after treatment with Watsu (p = 0.01) for SF-36 subscales of physical function, bodily pain, vitality and social function, but not for Aix. The repeated measures design with counterbalancing should reduce carryover effects although order effects may have occurred due to high dropout. In addition it used a volunteer sample.

Three studies had no separate control group, using a single group pretest-posttest design[25-27], limiting the validity of results. Lichtenberg et al's [27] pilot study of Shiatsu for schizophrenia showed significant improvements on scales relating to illness, psychopathy, anxiety, depression and others (p values ranged from 0.0015 to 0.0192). Brady et al [26] tested Shiatsu for lower back pain in 66 volunteers. Pain and anxiety significantly decreased after treatment (p < 0.001), which did not change when demographic variables were controlled for. Iida et al [25] investigated the relaxation effects of Shiatsu on anxiety and other side effects in 9 patients receiving cancer chemotherapy. The small and self-selected samples and lack of control group in these studies limits the quality and generalisablity of the results. In addition 13 of Brady et al's [26] participants had previously received Shiatsu

Long (2008) conducted a prospective observational study of 948 patients of Shiatsu practitioners in 3 different countries[7]. Significant improvement in symptoms, especially for tension or stress and structural problems (effect size 0.66 to 0.77) were demonstrated. This study is of greater quality than other Shiatsu studies as the sample size was powered and it used a longitudinal and pragmatic study design. For a longitudinal observational design, this study had a good response rate (67% of patients on average returned all questionnaires). Recruitment of patients was through practitioners, who received a rigorous training and kept a recruitment log. Confounding factors are reported and outcomes were accurately measured. However, data on non-respondents or those who refused to participate were not reported so evaluation of response bias is problematic.

Sundberg et al [19] and Ballegaard [21] used a pragmatic design - Shiatsu as part of an integrated model of healthcare or with other interventions (acupuncture and lifestyle adjustment). This reflects normal practice but specific effects of Shiatsu cannot be isolated.

There was insufficient evidence both in quantity and quality on Shiatsu in order to provide consensus for any specific health condition or symptom.

### Acupressure

Of a total of 71 included studies described as giving acupressure as an intervention, 2 were meta-analyses, 6 systematic reviews, 39 RCTs, five crossover trials, 5 within-subjects trials, 5 controlled non-randomised, 7 uncontrolled trials and 1 prospective study. These are summarised by health condition below.

### Pain

Pain was the most common issue addressed by acupressure studies and covered a range of topics. This included a systematic review, six RCTs with control groups and random assignment; 2 with non-randomised control groups or within-subject controls, and the remainder either did not have a control or random assignment. Overall, the evidence for the efficacy of acupressure for pain is fairly strong and can be graded as category 1 evidence. Although some studies had methodological flaws, studies consistently show that acupressure is more effective than control for reducing pain, namely dysmenorrhoea (acupressure at SP6) [9,28-30], lower back pain [31-33] and labour pain [34,35]. The evidence for minor trauma [36,37] and injection pain [38,39] is less conclusive and the evidence for headache is insufficient [40]. Each pain condition is discussed below.

#### Dysmenorrhoea

Of 4 papers for dysmenorrhoea, 1 was a systematic review 2 were RCTs, and one non equivalent control group. All studied school or university students, with sample sizes ranging from 30 to 216. Two used acupressure on SP6, The other used a combination of points. Both of the RCTs [28,30] compared acupressure to rest, which does not control for the placebo effect. Jun et al [29] compared acupressure to light touch, potentially controlling for non-specific effects but used sequential allocation which may create bias, although groups were homogenous in baseline demographics and dysmenorrhoea factors. All studies found a significant reduction in pain. Studies were generally good quality, with low attrition rates and validated measures (usually VAS). Only including students may limit generalisability and create Hawthorne bias. Acupressure procedure was generally well-reported; all studies reported 12 or 13 STRICTA items.

#### Labour pain

Two of the three studies of acupressure for labour pain were RCTs [34,35]. They both compared acupressure to touch, thus controlling for the effect of human touch; Chung et al [34] additionally had a conversation only control group. The third was a one group uncontrolled study [41]. Two studies usedLI4 [34,41]; Chung et al [34] additionally used BL67; Lee et al used SP6 [35]. All studies found acupressure significantly reduced pain,

#### Back and neck pain

Four studies on back or neck pain were identified, all RCTs and conducted by two groups of researchers, Hsieh et al [31,32] and Yip and Tse [33,42]. Hsieh et al unusually used a pragmatic design of four weeks of individualised acupressure compared to physical therapy. They also used powered samples, blinding where possible, valid outcome measures and intention to treat analysis to protect against attrition bias. A no treatment group was not included, limiting assessment of specific effects. Yip and Tse also compared acupressure to usual care, although an acupressure protocol was used. They also had powered sample sizes but no blinding. Comparison groups of aromatherapy and electroacupuncture, limit specific effects of acupressure. All four studies showed a significant reduction in pain.

#### Minor trauma

Two double-blind RCTs evaluated acupressure for minor trauma pain during ambulance transport [36,37]. Both used sham acupressure as a control, with Kober et al [36] additionally comparing to no treatment. Both studies showed significant reductions in pain, anxiety and heart rate. Limitations include fairly small sample and lack of no-treatment control.

#### Injection pain

Two studies evaluated acupressure for pain of injection [38,39]. Both studies showed reduction in pain but both were subject to limitations - Arai et al [39] only included 22 subjects although it was powered and randomised, with a sham treatment; Alavi et al's [38] trial was larger and randomised, but used a within-subjects crossover design which can create practice bias.

#### Headache

Only one study investigated headache [40], comparing a course of 8 sessions of acupressure to medication, which reduced pain. Although this used an RCT design, power calculation, intention-to-treat analysis, blinding and long follow up, there is very little detail on intervention (only 7 STRICTA items), randomisation, recruitment or limitations.

#### Dental pain

One RCT for dental pain [43] compared acupressure at LI4 to medication or sham acupressure, showing reduction in pain 4 and 24 hours after the first orthodontic treatment but not after second treatment. Although an RCT and well reported, only 23 patients completed the study, despite a power calculation specifying a sample of 156.

### Nausea & vomiting

Nausea and vomiting (N&V) was the second most commonly investigated health issue. The evidence was somewhat inconsistent and varied with type of nausea investigated. Post-operative nausea had strongest evidence, graded as Category 1 evidence mainly due to a Cochrane systematic review and update [8,44] and a meta-analysis [45]. The two systematic reviews [46,47] of chemotherapy-induced N&V give additional quality evidence, although little is true acupressure. Little reliable evidence is added by the RCT [48]. The three studies of acupressure for nausea in pregnancy are of variable quality. Although one has a small sample and uncontrolled study design [49], a well conducted RCT [50]and meta analysis [51] provide Category 2 evidence for nausea in pregnancy.

#### Post-operative

A Cochrane review [44] (update of a previous review [8]) and meta-analysis [45] indicate the extensive evidence for acupressure in treating postoperative N&V. All the studies in the review and the majority in the meta-analysis used acupoint PC6. The review concluded that acupressure reduced the risk of both N&V compared to sham, and reduced the risk of nausea but not vomiting compared to antiemetic medication. The meta-analysis concluded that all modalities of acupoint stimulation reduced postoperative N&V compared to control, and were as effective as medication. Both reviews were very high quality with comprehensive search terms and pooling of data.

#### Chemotherapy

Acustimulation, including acupressure, for nausea as a side-effect of chemotherapy also has been reported in a Cochrane review [46], as well as an RCT published subsequently [48] and a non-randomised trial [52]. Chao et al [47] also covered N&V as part of their review of adverse effects of breast cancer treatment.

The Cochrane review identified 11 trials and pooled data demonstrated significantly reduced vomiting but not nausea [46]. It was very good quality, with intention-to-treat analysis of pooled data and controlling for duplicate and language bias.

The RCT (n = 160)[48] was based on a pilot [53] included in the Cochrane review. It found significant reductions in delayed N&V but not acute N&V, results facilitated by the unusually long follow-up period. The main limitations are the lack of sample size calculation (despite conducting a pilot study) and patients breaking the blind.

The non randomised study [52] of self-acupressure on PC6 compared to anti-emesis medication found significant reductions in severity of N&V, duration of nausea and frequency of vomiting compared to control. However, these results are limited by a small and convenience sample.

#### Pregnancy

Three studies investigated N&V in pregnancy: one RCT [50]; one uncontrolled study [49] and one meta-analysis [51]. All used acupressure on PC6 (neiguan).

As concluded by the meta-analysis [51], the RCT found improvements compared to sham or control. Shin et al's RCT [50] is excellent quality with double-blinding, powered sample size, objective and subjective outcomes and good reporting. Markose et al [49] also found improvements in nausea, vomiting and retching, but due to lack of control group, small sample, high attrition and poor reporting the evidence is limited.

The meta-analysis included studies on all forms of acustimulation and was generally well conducted, although it did not attempt to find unpublished material and only 3 databases were used.

### Renal disease

Five papers (based on four RCTs) investigated the use of acupressure for symptoms of renal disease. Due to limitations, repeated in all studies due to the common research team, evidence is category 2. Three compared acupressure to sham points/electrical stimulation and to usual care [54-56], the fourth to usual care only [57]. The studies used different points for different symptoms, including fatigue [55,57], depression [56,57] and sleep [54,56]. All studies showed improvements compared to control but also found improvements in the sham/electrical stimulation group compared to control, suggesting that the effects of acupressure on these symptoms are non-specific. Sample sizes were between 62 (powered) and 106 and had low attrition rates. One study used blinding [54], the others may have been subject to placebo or observer bias. Between 9 and 15 STRICTA items were reported and interventions and outcome measures were validated.

### Sleep and alertness

Five studies investigated acupressure for sleep in elderly long term care facilities [58-62], and one investigated alertness in the classroom [63]. Evidence for improving sleep quality in institutionalised elderly is consistent from a number of high quality studies and is category 1. Four of the sleep studies were RCTs [59-62], an additional single-group pilot study of only 13 people contributes little to the evidence base [29]. The four RCTs all used different acupoints. Two compared acupressure to sham points and control (conversation [62]or routine care [60]) but only one found significant improvements in sleep for acupressure compared to sham [62], giving limited evidence for specific effects. Three of the studies had powered and randomly selected samples (between 44 and 246) [60,62], validated procedure [62], intention-to-treat analysis or triple blinding [60].

The one study on alertness in the classroom [63]was a crossover study, randomly assigning 39 students to either stimulation-relaxation-relaxation or relaxation-stimulation-stimulation. Compared to relaxation, stimulation acupressure improved alertness. Although students were blinded, the majority correctly discerned the treatment. This did not significantly affect the results, although it raised p to 0.0484. Potential Hawthorne effect, small sample size (39) and low generalizability reduce the quality. Crossover design should reduce effects of retesting, carryover or time-related effects, although practise effect may be present (especially with self-report).

### Mental health

Five studies investigated mental health, specifically dementia [64,65] and stress or anxiety [66-68]. The quality was very variable, with two pilot studies with sample sizes of 12 and 31 [64,68], a small one group study of 25 women [67] and two larger RCTs [65,69]. Category 2 evidence was present for anxiety related to surgery, although this was compared to sham only[69]. Fairly good evidence existed for agitation in dementia compared to control, although generalisability was limited by small sample size, lack of control and high attrition[65]. Evidence for reducing stress, anxiety and heart rate and thus enhancing spontaneous labour is promising, but limited by lack of control and a small, volunteer sample [67].

### Chronic respiratory conditions

Six studies on respiratory conditions were identified, chronic obstructive pulmonary disease (COPD)[70-73], chronic obstructive asthma [74] and bronchiectasis [75]. Overall, the evidence is Category 2, as studies were well designed but had a number of methodological flaws. Study designs included two controlled trials using randomised blocking design, matching groups for demographic and clinical factors [71,72]; one crossover design [70]; two pilot RCTs [74,75] and an RCT [73]. Results showed improvements in dyspnoea and decathexis compared to sham, although limited by high attrition, poor blinding and a small sample [70]. The pilot studies (with the same authors) showed improved quality of life for asthma patients [74] and sputum and respiratory scores for bronchiectasis compared to control [75], but are limited by small sample sizes, high dropout and lack of blinding. The matched studies [71,72] provided high quality evidence for improvements in dyspnoea and related outcomes, with valid and reliable interventions and outcome measures, and blocking design giving more powerful treatment effects for small samples.

### Anaesthesia/consciousness

Three studies investigated the effects of acupressure on levels of anaesthesia or consciousness. These levels include the acoustic evoked potential (AEP), changes in which reflect the depth of anaesthesia and transition from awake to anaesthetised [76]; bispectral index (BIS) and spectral edge frequency (SEF) which are measures of the level of consciousness during anaesthesia/sedation [77,78]. Overall, the evidence is Category 3 as only three studies were identified, all had repeated measures designs and small sample sizes (between 15 and 25), although one was powered [68,76-78]. Patients acting as their own controls in these studies can cause practice and carryover effects, although reduced by counterbalancing/randomising of treatment order. However, lack of control group and lack of details on sample selection limit the evidence.

### Stroke

Three studies investigated acupressure for stroke [79-81]. All three were RCTs; Shin and Lee [80] used a blocked randomised design comparing acupressure to acupressure plus aromatherapy, Kang et al [81] randomised to acupressure or control groups; McFadden and Hernandez [79] used a crossover design comparing acupressure to control. Although studies used good designs and results suggested significant improvements in pain[80], motor power [80], limb function [81], daily living[81], depression [81], and heart rate [79], all findings were limited by small unpowered samples and poor reporting, so evidence is rated at Category 2.

### Body weight

Two randomised studies investigated the effect of acupressure on body weight, although for very different conditions - weight loss [82] and weight gain in premature babies[83]. Elder et al's [82] RCT compared 'Tapas Acupressure Technique'^® ^(TAT)^1^, qi gong and control (self directed support). TAT resulted in greater weight loss than both qi gong and control. Chen et al's[83] RCT compared acupressure and meridian massage to routine care, resulting in significantly more weight gain. The weight-loss study was high quality with a large sample, design-adaptive group allocation (equivalent to randomisation, but balanced for demographic and clinical factors). The weight gain study was randomised and matched for weight and gestation age and used blinding (although details are not clear), but had a small sample size and lack of information on randomisation, allocation, drop outs, harms and ethics. The evidence for weight loss/gain is Category 2 as more studies are needed.

### Visual impairment

Two non-randomised studies from China and Taiwan evaluated acupressure for schoolchildren with visual impairment [84,85]. Both found improvements compared to control but were limited in reporting of study design and findings and did not randomise. With only 2 studies, both with significant limitations, the evidence for acupressure for improving eyesight is Category 3.

### Other conditions

The remaining 11 articles on acupressure investigated distinct health conditions which could not be grouped.

A systematic review evaluated the effect of acupoint stimulation for side effects of breast cancer treatment[47]. 26 studies were identified, concluding that evidence is high quality for nausea and vomiting but weak for all other adverse effects. It was well conducted with appropriate inclusion criteria, Jadad scale for rating and two independent raters.

Ballegaard et al [86,87] studied acupressure for angina. The 1999 study [86] was a cost benefit analysis and used non-equivalent control groups, a volunteer and convenience sample and used co-interventions of acupuncture and the self-care program. The 2004 study [87] had a good sample size although subjects were not randomised, the follow-up period was long, but no equivalent control group or blinding. Again, it was difficult to isolate the effects of acupressure from co-interventions. At baseline the sample did not significantly differ to Scandinavian heart patients. This 'quality control review', is subject to selection, expectation and social biases.

Gastrointestinal motility was studied by Chen et al [88,89], with significant improvements demonstrated. In [88], although the intervention was well reported, randomisation is not described (although groups were homogenous for a range of variables). In [89] the sample was small and not powered and the study was single-blind, although groups were homogenous. Significant effects were observed.

A poorly reported study observed that acupressure on PC6 significantly reduced gagging in 109 dental patients [90]. The study was described as double-blind although blinding procedures were not described. Details of the sampling were not available.

In a comparison of acupressure with oxybutinin for nocturnal enuresis in children[91], the main flaw was the very small sample size, with no details of sampling, comparison of groups or randomisation, potential selection bias and no placebo/sham group.

A controlled trial of acupressure for 30 patients with peripheral arterial occlusive diseases (PAOD) reported a significant reduction in transcutaneous oximetry[92]. This is a poor quality study with an apparent lack of randomisation and non-equivalent control group, poor reporting and no comparison of groups, although outcomes are objective and intervention is well reported.

A high quality RCT of acupressure for symptoms of diabetes found improvement in Hyperlipidemia, hypertrophy and kidney function [93] Acupressure was given regularly for 3 years, an unusually long follow up period and showed improvements in hyperlipidemia, ventricular hypertrophy, kidney function and neuropathy. The sample size was appropriate (although fairly high attrition) and group allocation was random. Very good description of treatment was provided (14 STRICTA items reported) although discussion is limited.

Yao et al [94] conducted a single group study of massage combined with acupressure for 85 patients with chronic fatigue syndrome. Treatment was effective in 91.8% of cases. This study did not use any clear outcome measures, had no control, and only reported 7 STRICTA items, and given its poor reporting it is low quality.

An uncontrolled pilot study was conducted of vaginal acupressure for sexual problems[95]. This showed significant improvements in symptoms, physical health, mental health, sexual ability and quality of life. This study is severely limited by small sample, lack of control, no details of recruitment, unvalidated and subjective outcome measures and poor reporting of acupressure. In addition the intervention did not appear to be based on meridian theory.

Sugiura et al [96] conducted an uncontrolled study with 22 healthy volunteers of the effects of acupressure on yu-sen, souk-shin and shitsu-min on heart rate and brain activity. Heart rates decreased. This study investigated mechanisms rather than effectiveness.

### Analysis/Summary of quality

**Figure 2:**
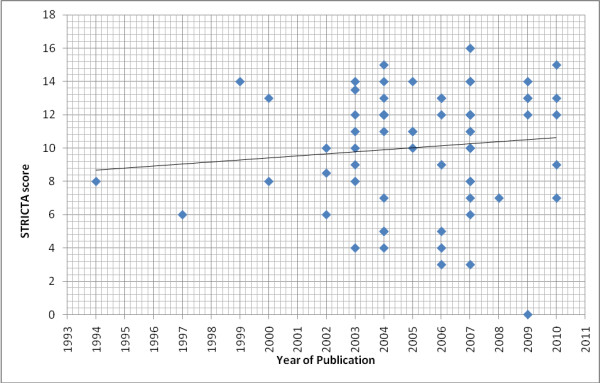
**STRICTA scores over time**.

**Figure 3:**
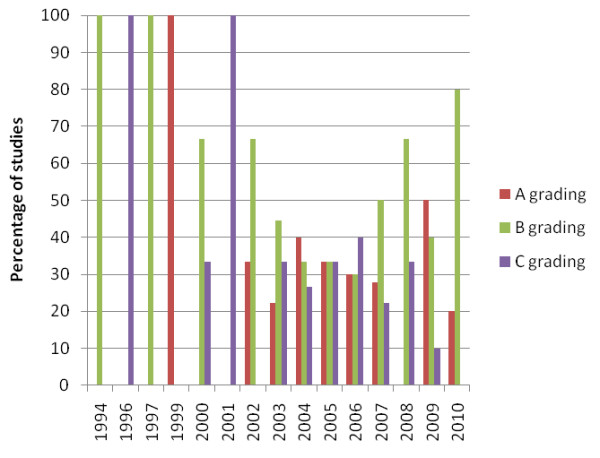
**Chart of study quality over time**.

**Figure 4:**
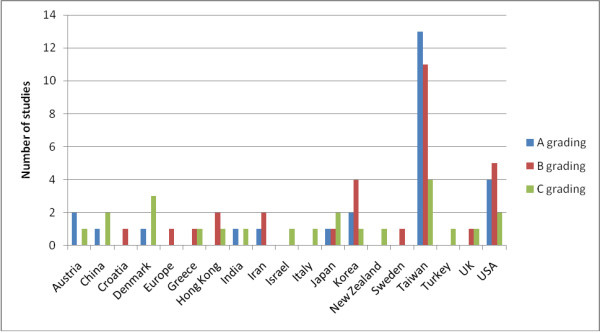
**Country of study**.

Twenty-two of the 80 included studies were graded C (the lowest quality grading). All five of the studies in Chinese language were graded C (or ungraded), and most of the Shiatsu studies were graded C. Analysis of results over time suggests some improvement in the evidence base. Figure 2 shows an improvement in the average number of STRICTA items reported by studies, shown by the line of best fit. Figure 3 indicates a reduction in the percentage of C graded papers over time, and an increase in those graded B. Figure 4 shows the numbers of studies and numbers of studies for each A/B/C grading for the different countries. This shows no obvious trend, although countries publishing more studies (Taiwan, USA and Korea) seem to have better quality studies, compared to countries with only one or two publications. Regarding quality appraisal, in a third of papers, a third reviewer was need to reach agreement on quality grading.

## Discussion

### Summary of evidence

These findings provide an important addition to the existing knowledge base on Shiatsu but are very limited in providing any evidence of efficacy for Shiatsu. To our knowledge this is the first systematic literature review for shiatsu.

The strongest evidence for acupressure was for pain, post-operative nausea and vomiting, and sleep.

#### Study design & quality

While much of the research is of insufficient quality to provide consensus on Shiatsu or acupressure use, some high quality clinical research (particularly around pain) does exist. The methodological limitations of the studies reported in this systematic literature review included small sample sizes, non-reporting of follow up, insufficient details on sampling, high drop-out rates, uncontrolled design and lack of blinding. Many studies were also underpowered.

Although most studies were RCTs, many studies used a controlled design but controls were non-randomised (8), crossover (5) or within-subjects (6) or they were uncontrolled (10), or observational (1). Lack of randomisation, allocation concealment and comparable treatments can create bias as non-randomised controlled trials can be subject to confounding factors such as time-related or seasonal bias. Evidence for Shiatsu is thus severely limited as only 3 of the 9 studies used a control group, one of which was non-random, with two pilot studies. Crossover designs may be subject to practice effect, especially for self-administered acupressure. Within subjects repeated measure designs can also be subject to learning, and are only useful for stable populations such as those with a chronic disease or healthy volunteers (as used by studies on anxiety, dementia and consciousness in this review). One-group uncontrolled studies are of limited value due to a range of potential confounding variables. Longitudinal designs such as [7] are useful to evaluate effects of a treatment, but again causality cannot be implied, and there is increased risk of Hawthorne effect or conditioning. Well-conducted randomised trials are therefore more likely to have internal validity and thus accurately estimate the causal effects of interventions than non-randomised studies [15]. However, certain study designs are more appropriate for certain interventions and populations[97] and contention is emerging about how complementary medicine should be evaluated[98-103]. The complexity of interventions such as Shiatsu, including their patient-centred and individualised nature, practitioner and non-specific effects, the influence of patient choice, and potential synergistic effects require innovative evaluative approaches.

Most studies used a small number of acupoints for a specific condition or symptom in a protocol approach, which facilitates replicability[104]. MacPherson et al [105] identify three levels of individualisation in acupuncture: "explanatory" trials which use the protocol approach; partially individualised treatments using some fixed points plus some flexible point choice; and "pragmatic" trials which use fully individualised treatment unique for each patient, as used in Shiatsu/TCM treatment[105]. Pragmatic trials can be highly valuable, for example the trial of acupuncture for back pain which informed NICE clinical guidance in the UK[106].

There was an improvement in the quality/reporting of papers over the time period searched. This may have been due to a greater appreciation of research amongst practitioners, advances in research methods in acupressure/shiatsu and the recent publication of a number of guidelines on presenting research such as the CONSORT, STRICTA and TREND statements used in this review [11,12,14].

The reporting of studies was very limited for many papers, with items most commonly missing from the CONSORT checklist including: 1a (identification as RCT in title); 16 (numbers of participants included in each analysis); 6b (changes to trial outcomes); 8,9 and 10 (details of randomisation procedure); 14b (why the trial was ended); and 23 and 24 (registration number and full protocol access) [12]. The average of 10.09 (63%) of applicable STRICTA items reported is similar to a previous review (53.4%) [107]. The increase in the number of STRICTA items reported over time is likely due to the gradual adoption of the STRICTA guidelines published in 2001 [11,107]. In common with this previous review the items most commonly missing were details of practitioner background, setting/context and explanations to patients, as well as amount of pressure used (equivalent to depth of insertion of needle), style of acupressure, *de qi *or the extent treatment was varied, perhaps less relevant to acupressure than acupuncture. Awareness of STRICTA guidelines is likely to be the key factor[107].

### Implications for practice

#### For conventional practitioners

Many of the conditions with the strongest evidence (pain, post-operative nausea and vomiting, and sleep) are side effects of or challenging symptoms for conventional medicine suggesting that an integrated treatment approach may be of benefit. Conventional healthcare practitioners may therefore consider acupressure, in particular: SP6 for dysmenorrhoea; PC6 for N&V postoperatively, in chemotherapy and pregnancy; combinations of ST36, SP6, KI1, KI3, HT17, KI11 and GB34 for renal symptoms; a range of points for COPD; HT7 and other points for sleep in elderly residents; and perhaps GB20, GV20, HT7, PC6 and SP6 for agitation in dementia. The evidence for protocol-based treatment supports suggestions that nurses incorporate acupressure and Shiatsu into their practice, in particular for pain relief, fatigue in cancer, augmenting effects of medication, providing comfort and improving breathing [108-110]. Shiatsu could be effectively delivered in general practice but further research in clinical and cost effectiveness is warranted [111].

#### For shiatsu/CAM practitioners

While much of the research carried out with Shiatsu or acupressure as an intervention is of insufficient quality to inform practice, the high quality evidence for pain, post-operative nausea and vomiting, and sleep may be of use to Shiatsu and acupressure practitioners. These symptoms highlight the value of acupressure/Shiatsu as a complementary approach

to conventional treatment. The findings relating to protocol-based acupressure may not directly inform the evidence base for more individualised and holistic treatments. However, the evidence for a specific acupoint for a specific symptom/condition can be integrated into an individualised treatment by combining with points suited for the individual. Hsieh et al provide pragmatic evidence for individualised treatment for low back pain and headache [31,32,40]. Some studies also supported the long-term effects of acupressure/Shiatsu, for example for headache [40], low back pain [31,32], and nausea and vomiting [48].

This review has highlighted the contention around the specificity of CAM treatments. Acupressure was often effective compared to control but not sham or medication, suggesting that effects are non-specific. Examples include labour pain [34], dysmenorrhoea [112], renal symptoms of fatigue, depression and sleep [54-56,59] and nausea and vomiting [8]. However, other studies found effects compared to sham treatment for similar conditions [8,35-37,47,62], and patient's belief in treatment may not affect results [63], suggesting specific effects. This review therefore provides little clarity on specificity of effects.

Shiatsu is an inherently safe treatment [113]. Four single case reports of adverse events occurring following Shiatsu massage were identified (not included in review)[114-117] as this review focussed on efficacy rather than safety these findings were incidental and there are likely to be more reports on safety. This is an important area for the profession regarding safety issues and possible causal links between Shiatsu and adverse events. Professional bodies for Shiatsu may need to consider the development and piloting of an adverse event reporting system for Shiatsu. Work by Andrew Long provides a useful typology of adverse effects [118]. These are: Type 1: Responses unconnected to the CAM modality; Type 2: Transitional effect (client-perceived and theory-consistent); Type 3: Transitional effect (theory and experientially consistent); Type 4: Undesired, but not unsafe event or effect; Type 5: Potentially adverse event or effect and possible risk to client safety. This typology could be utilised in future studies.

### Implications for research

The research base for Shiatsu is still very much in its infancy and the profession will need to work closely with practitioners and researchers in order to build up a larger body of evidence. Given the prevalence of Shiatsu used in the UK (820 registered practitioners/teachers/trainee teachers^2^), the need for high quality research is imperative. Shiatsu practitioners should be encouraged to engage in research using well designed and reported studies, in particular with large samples and controlled designs.

Results have highlighted that alternative RCT designs may be necessary, such as:

• Whole systems research, which includes qualitative and quantitative methods to include the broader aspects of treatment, not just the intervention[119,120]

• Mixed-methods research, as qualitative data can provide additional information on patients' and/or practitioners' views on the effectiveness of treatment. Many studies are including such qualitative data as part of their design to provide a broader picture of patient outcomes [119].

• Preference trials, which include patient choice of treatment, often important in CAM, producing more generalisable results, such as in the study by Lucini [20],

• Early phase research or pilot studies to generate hypotheses, identify the most appropriate health conditions, patient groups and treatments to test in full clinical studies[121], given the limited evidence base for Shiatsu.

• A pragmatic design as used by some studies in this review. Pragmatic trial design overcomes some of the barriers of conducting RCTs in CAM, including improved recruitment and providing patient-centred treatment as usual. Only six studies used a pragmatic design; three for shiatsu [7,19,86] and three for acupressure [31,32,87]. Examples of pragmatic trials are the cohort multiple randomised controlled trial [122] and health services research [101]. There is promising research using both a pragmatic approach to evaluate Shiatsu as part of an integrated or massage intervention [19,21,123]. A flexible protocol approach could be used to improve replicability[104].

• One of the main issues in RCTs of complementary approaches is the control treatment, for example the limitations of blinding and sham acupressure. The included studies have confirmed that "sham" acupressure including light touch at acupoints does have an effect. The highest quality evidence was from three armed trials which use sham treatment and an inert control, as advocated in acupuncture research[124]. Shiatsu (as distinct from acupressure) presents further complexities as treatments are based on Hara diagnosis and rarely if ever "standardised". This needs to be adequately reported in papers, following guidelines such as CONSORT or TREND.

Although excluded from this review due to resource constraints, qualitative studies provide additional information on patients' and/or practitioners' views on the effectiveness of treatment [125-127]. Many studies now include such qualitative data as part of their design to provide a broader picture of patient outcomes.

Particular areas to focus research, commonly treated with Shiatsu/acupressure include psychological and musculoskeletal conditions, in particular neck/shoulder, lower back problems, arthritis, depression, stress and anxiety[6]. There is also good evidence for sleep and symptoms of renal disease, but studies to increase the generalisability of these findings is necessary.

Taiwanese researchers appear to have been most prolific in this area, as well as Korea and the USA. Given the increasing use of CAM in Europe more research based in European countries may be needed. Given the prevalence of Shiatsu used in the UK, the need for research is imperative.

Use of quality guidelines such as STRICTA and CONSORT is advised to improve the reporting of studies, especially details of interventions, to provide replicability as well as to inform practice [11].

### Strengths and limitations

A wide range of databases was used to maximise the number of articles captured. This review used recognised quality checklists to evaluate studies and each was independently assessed by 2 reviewers, with fairly high inter-rater agreement, and with a third reviewer for adjudication.

The checklists used to calculate the quality of the reporting of studies (CONSORT, TREND etc) were useful but do have limitations. In particular with such a broad range of study designs other than RCTs, the appropriateness of checklists for specific study designs is limited. For example the TREND checklist for nonrandomised study designs may require additional specific criteria for specific types of nonrandomised designs [14].

Searches were restricted to UK/USA databases due to resource constraints; including Asian databases may have added to the evidence. Language bias may also have been present, although some Chinese language studies were included. There was no attempt to find grey literature except searching for UK postgraduate theses; no contact was made with individual authors due to the large numbers of authors.

As this review was not limited by health condition, the breadth of the included studies necessitated limiting inclusion by excluding studies prior to 1990. This may have created bias.

As the quality assessment in a systematic review depends on contextual and pragmatic considerations, it was necessary to limit the number of articles reviewed due to time and resource constraints [97]. In particular, purely qualitative studies were excluded, which may have limited results given the now recognised value given to qualitative outcome measures, particularly in complex interventions such as Shiatsu.

## Conclusions

This review identified very little Shiatsu research, suggesting well designed studies are needed. The evidence for acupressure and pain is generally consistent and positive. There is also evidence for acupressure improving sleep in institutionalised elderly. Acupressure studies for nausea and vomiting have been somewhat inconsistent, with strongest evidence for post-operative nausea, and may merit further research. Evidence for pain, nausea and vomiting and sleep support an integrated approach using acupressure for conditions problematic to conventional medicine. There is limited evidence for chronic respiratory conditions, especially COPD, and psycho-social aspects of health, anaesthesia and other health conditions. Evidence on specific vs non-specific effects is inconclusive. This review highlighted the challenges of conducting rigorous research into CAM, which are complex, individualised and patient-centred, but illustrates useful study designs such as pragmatic/flexible protocol, 3 armed with sham and no treatment, longitudinal and preference trials. Evidence appears to be improving in quantity, quality and comprehensive reporting, but there is still much room for improvement.

## Competing interests

The authors declare that they have no competing interests.

## Authors' contributions

XL conducted the searches and retrieved the articles. XL and AL reviewed the articles and NR was the adjudicator. XL and AL compiled the evidence tables. AL and NR wrote the introduction and discussion section. AL created the tables and graphs in the main text. All authors read and approved the final manuscript.

## Endnotes

1. http://www.tatlife.com/

2. Personal correspondence with Shiatsu Society UK

## Pre-publication history

The pre-publication history for this paper can be accessed here:

http://www.biomedcentral.com/1472-6882/11/88/prepub

## Supplementary Material

Additional file 1 **Table 1**. This table contains details of each of the included studies Click here for file
