# Supplementary material for: The evidence for Shiatsu: a systematic review of Shiatsu and acupressure
Source: BMC Complement Altern Med. 2011 Oct 7;11:88. doi: 10.1186/1472-6882-11-88 (PMC3200172; doi:10.1186/1472-6882-11-88)
Supplement: Additional file 1 — Table 1. This table contains details of each of the included studies [file 1472-6882-11-88-S1.DOC]

| **Shiatsu** | | | | | | | | | | |  |
| --- | --- | --- | --- | --- | --- | --- | --- | --- | --- | --- | --- |
| **Reference** | **Design** | **Total sample** | **Intervention** | **Administration** | **Results** | **Reporting** | | | **Grading[[1]](#footnote-2)** | |  |
|  |  |  |  |  |  | **Checklist** | **score** | **% score*** | |  |  |
| Angina | | | | | | | | | | |  |
| Ballegaard et al 1996 | Controlled trial | 69 angina patients | Integrated treatment with shiatsu on CV17 | Self-administered | Incidence of death/MI reduced to 7% (compared to between 15% and 21%) | TREND | 22 | 41.51 | | C |  |
| Back/neck pain | | | | | | | | | | |  |
| Sundberg et al 2009 | RCT | 80 primary care patients | Integrated care, one of which was shiatsu | Shiatsu practitioner | No significant effects | CONSORT | 24 | 64.86 | | B |  |
| Brady et al 2001 | Single group | 66 Shiatsu patients (private practice) | Individualised shiatsu | Shiatsu practitioner | Pain decreased (p<0.001), anxiety reduced (p<0.0001) | TREND | 34 | 64.15 | | C |  |
| Fibromyalgia | | | | | | | | | | |  |
| Faull 2005 | Repeated measures | 17 volunteers with fibromyalgia | Watsu massage | Watsu practitioner | SF36 improvements in subscales of physical function, bodily pain, vitality,social function (p=0.01) | TREND | 29 | 54.72 | | C |  |
| Cancer | | | | | | | | | | |  |
| Iida et al 2000 | One group | 9 cancer patients | Shiatsu massage | n/s | Reduced anxiety for group with strong anxiety (p=0.09), increased relaxation for group with weak anxiety (p=0.01) | TREND | 16 | 30.19 | | C |  |
| Inducing labour | | | | | | | | | | |  |
| Ingram et al 2005 | Controlled audit | 142 pregnant women | Shiatsu on points GB21, LI4 and SP6; breathing techniques and exercises | Self administered (taught by midwife) | Labour more likely to be spontaneous (p=0.038) and longer (p=0.03). | TREND | 28 | 52.83 | | C |  |
| Mental health | | | | | | | | | | |  |
| Lichtenberg et al 2009 | One group | 12 schizophrenic inpatients | Individualised Shiatsu | Shiatsu practitioners | Improved on a range of outcomes p values ranged from 0.0015 to 0.0192 | TREND | 21 | 39.62 | | C |  |
| General (range of conditions) | | | | | | | | | | |  |
| Long 2008 | Uncontrolled | 948 Shiatsu patients (private practice) | Routine practice | Shiatsu practitioner | Symptom scores improved significantly over the 6 months | N/A | | | B | |  |
| Chronic stress | | | | | | | | | | | |
| Lucini et al 2009 | Controlled nonrandomised | 70 patients (source not described) | Individualised Shiatsu | Shiatsu practitioner | Reduced stress compared to control (p=0.032) | TREND | 28 | 52.83 | | B | |
| n/s: not specified | | | | | | | | | | |  |

| **Acupressure** | | | | | | | | | | | | | | |
| --- | --- | --- | --- | --- | --- | --- | --- | --- | --- | --- | --- | --- | --- | --- |
| **Pain: Category 1** | | | | | | | | | | | | | | |
| **Dysmenorrhoea (4 papers)** | | | | | | | | | | | | | | |
| **Reference** | **Design** | **Total sample** | **Intervention** | **Administration** | **Results** | **Reporting** | | | | | | | **Grading[[2]](#footnote-3)** | |
|  |  |  |  |  |  | **Checklist** | **score** | | **% score*** | | **Stricta score** | |  | |
| Cho and Hwang 2009 | SR | 30 studies | Acupressure | N/A | Acupressure alleviates menstrual pain | CASP | 5 | | 48.6 | | N/A | | A | |
| Chen and Chen 2010 | RCT | 134 students | 4 groups. ST36;CO4; CO4-SP6Matched Points; Control | researcher | matched points significantly reduced pain (p=0.02), distress (p=0.001) and anxiety (p=0.001) compared to control, after six months. CO4 (p=0.02) reduced pain but not distress and anxiety. ST36 had no significant effects. | CONSORT | 22 | | 56.8 | | 12 | | A | |
| Wong et al 2010 | RCT | 40 students | Acupressure on SP6 | Self | Pain reduced (p=0.003; p=0.012; p=0.024) | CONSORT | 15 | | 48.6 | | 13 | | B | |
| Jun et al 2006 | Controlled non randomised | 58 students | Acupressure on SP6 compared to light touch | researcher | Dysmennorhea severity reduced post-treatment (p=0.000) and for up to 2h (p=0.032) | TREND | 37 | | 48.6 | | 12 | | B | |
| **Labour pain (3 studies)** | | | | | | | | | | | | | | |
| **Reference** | **Design** | **Total sample** | **Intervention** | **Administration** | **Results** | **Reporting** | | | | | | | **Grading** | |
|  |  |  |  |  |  | **Checklist** | | **score** | | **% score*** | | **Stricta score** |  | |
| Chung et al 2003 | RCT | 127 parturient women | Acupressure on LI4 and BL67 compared to light touch and control | midwife (trained) | Decreased pain compared to control (p=0.017) | CONSORT | | 15 | | 40.5 | | 13.5 | A | |
| Lee et al 2004 | RCT | 75 women in labour | Acupressure on SP6 compared to light touch | researcher | Pain reduced post treatment (p=0.012), 30mins later (p=0.021) and 60mins later (p=0.012) | CONSORT | | 13 | | 35.1 | | 14 | A | |
| Waters and Raisler 2003 | One group | n/s | Ice acupressure on LI4 | n/s, probably midwife | Reduced pain | CONSORT | | 29 | | 78.4 | | 8 | B | |
| **Back/neck pain (4 studies)** | | | | | | | | | | | | | | |
| **Reference** | **Design** | **Total sample** | **Intervention** | **Administration** | **Results** | **Reporting** | | | | | | | **Grading** | |
|  |  |  |  |  |  | **Checklist** | | **score** | | **% score*** | | **Stricta score** |  | |
| Hsieh et al 2004 | RCT | 146 chronic low back pain patients | Individualised acupressure compared to physical therapy | physical therapist trained in acupressure | Pain reduced posttreatment (p = 0.0002) and after 6months (p = 0.0004). | CONSORT | | 25 | | 67.6 | | 5 | B | |
| Hsieh et al 2006 | RCT | 129 chronic low back pain patients | Individualised acupressure compared to physical therapy | acupressure therapist | Disability decreased (CI = 5.7 to -1.9). Also reduced pain | CONSORT | | 26 | | 70.3 | | 4 | A | |
| Yip and Tse 2004 | RCT | 61 adults with sub acute or chronic low back pain | acupressure on BL22,23,25,40 compared to electrical stimulation and control | nurse trained in TCM | Reduced pain compared to control (p=0.0001) | CONSORT | | 21 | | 56.8 | | 13 | B | |
| Yip and Tse 2003 | RCT | 28 adults with neck pain | Acupressure on 20 points compared to control | nurse trained in TCM | Reduced pain (p=0.02) | CONSORT | | 19 | | 51.4 | | 13 | B | |
| **Minor trauma (2 studies)** | | | | | | | | | | | | | | |
| **Reference** | **Design** | **Total sample** | **Intervention** | **Administration** | **Results** | **Reporting** | | | | | | | **Grading** | |
|  |  |  |  |  |  | **Checklist** | | **score** | | **% score*** | | **Stricta score** |  | |
| Lang et al 2007 | RCT | 32 radial fracture patients | Acupressure on GV20 and LR4 compared to sham | paramedic | Lower pain (p=0.001) and anxiety (p=0.022) and heart rate (p<0.05) | CONSORT | | 22 | | 59.5 | | N/A | B | |
| Kober at al 2002 | RCT | 60 minor trauma patients | Acupressure on LI4, CV9, PC66, BL60, GV20 compared to sham and no treatment. | paramedic | Less pain, anxiety and heart rate (p<0.01) | CONSORT | | 18 | | 48.6 | | 8.5 | B | |
| **Injection (2 studies)** | | | | | | | | | | | | | | |
| **Reference** | **Design** | **Total sample** | **Intervention** | **Administration** | **Results** | **Reporting** | | | | | | | **Grading** | |
|  |  |  |  |  |  | **Checklist** | | **score** | | **% score*** | | **Stricta score** |  | |
| Arai 2008 | RCT | 22 healthy females | Acupressure on Extra1 compared to sham | Not clear (probably researcher) | Pain reduced (p=0.006) | CONSORT | | 13 | | 35.1 | | 7 | B | |
| Alavi 2007 | Crossover trial | 64 patients receiving penicillin injections | Acupressure on BL31 compared to none | nurse specially trained | Pain reduced (p<0.000) | CONSORT | | 16 | | 43.2 | | 11 | B | |
| **Headache (1 study)** | | | | | | | | | | | | | | |
| **Reference** | **Design** | **Total sample** | **Intervention** | **Administration** | **Results** | **Reporting** | | | | | | | **Grading** | |
|  |  |  |  |  |  | **Checklist** | | **score** | | **% score*** | | **Stricta score** |  | |
| Hsieh et al 2010 | RCT | 28 outpatients with chronic headache | Acupressure (points NS) | n/s probably conventional practitioner | Pain decreased compared to medication post treatment and month follow up(p=0.047 and p=0.002) | CONSORT | | 25 | | 67.6 | | 7 | B | |
| **Dental (1 study)** | | | | | | | | | | | | | | |
| **Reference** | **Design** | **Total sample** | **Intervention** | **Administration** | **Results** | **Reporting** | | | | | | | **Grading** | |
|  |  |  |  |  |  | **Checklist** | | **score** | | **% score*** | | **Stricta score** |  | |
| Salam 2008 | RCT | 36 dental patients aged 11 to 16 | self-administered acupressure at LI4 compared to sham or medication control | Self | After first visit, back teeth pain and pain affecting diet were worst in control (p=0.013 and p=0.021) | CONSORT | | 24 | | 64.9 | | 10 | C | |
| **Nausea and vomiting: Category 1 and 2** | | | | | | | | | | | | | | |
| **Post-operative** (PONV) **(2 reviews) Category 1** | | | | | | | | | | | | | | |
| **Reference** | **Design** | **Total sample** | **Intervention** | **Administration** | **Results** | **Reporting** | | | | | | | **Grading** | |
|  |  |  |  |  |  | **Checklist** | | **score** | | **% score*** | | **Stricta score** |  | |
| Shiao and Dune 2006 | Meta-analysis | 72 studies | Acustimulation | N/A | Acustimulation as effective as medications | CASP | | 8 | | 80.0 | | N/A | A | |
| Lee and Fan 2009 | SR | 40 studies | Acustimulation of PC6 | N/A | Prevented PONV | CASP | | 8 | | 80.0 | | N/A | A | |
| **Pregnancy (3 studies) Category 1** | | | | | | | | | | | | | | |
| **Reference** | **Design** | **Total sample** | **Intervention** | **Administration** | **Results** | **Reporting** | | | | | | | **Grading** | |
|  |  |  |  |  |  | **Checklist** | | **score** | | **% score*** | | **Stricta score** |  | |
| Helmreich et al 2006 | Meta-analysis | 13 studies | Acustimulation | N/A | Acupressure had a greater impact than acupuncture (p<0.001). | CASP | | 5 | | 50 | | N/A | B | |
| Shin et al 2007 | RCT | 66 women with hyperemesis gravidarum | Acupressure on PC6 compared to placebo and control | Researcher | Nausea and vomiting lower (p<0.05) | CONSORT | | 18 | | 48.6 | | 11 | A | |
| Markose et al 2004 | One group | 35 pregnant women | Acupressure on PC6 | Self | Reduced nausea (p=0.008), vomiting (p=0.000), retching (p=0.004) and distress (p=0.002), | TREND | | 15 | | 28.3 | | 4 | C | |
| **Chemotherapy (3 papers) Category 2** | | | | | | | | | | | | | | |
| **Reference** | **Design** | **Total sample** | **Intervention** | **Administration** | **Results** | **Reporting** | | | | | | | **Grading** | |
|  |  |  |  |  |  | **Checklist** | | **score** | | **% score*** | | **Stricta score** |  | |
| Ezzo et al 2006 | SR | 11 studies | Acustimulation | N/A | Acupressure reduced mean acute nausea severity (SMD = -0.19; 95% confidence interval -0.37 to -0.01; P = 0.04) | CASP | | 9 | | 90.0 | | N/A | A | |
| Dibble et al 2007 | RCT | 160 breast cancer patients | Self administered PC6 acupressure compared to sham and control | Self | Significantly reduced delayed nausea and vomiting compared to placebo (p=0.002; p<0.006 respectively) and usual-care groups (p<0.0001; p=0.006) | CONSORT | | 20 | | 54.1 | | 15 | B | |
| Shin et al 2004 | Non equivalent control group | 40 gastric cancer patients | Acupressure on PC6 compared to medication | Self | Reduced severity of nausea and vomiting, duration of nausea, frequency of vomiting (all p<0.01). | TREND | | 28 | | 52.8 | | 7 | B | |
| **Sleep and alertness** (6 studies) **Category** **1** | | | | | | | | | | | | | | |
| **Reference** | **Design** | **Total sample** | **Intervention** | **Administration** | **Results** | **Reporting** | | | | | | | **Grading** | |
|  |  |  |  |  |  | **Checklist** | | **score** | | **% score*** | | **Stricta score** |  | |
| Hsu et al 2006 | RCT | 50 | HT7 acupressure compared to light touch | Nurses trained for project | Improved sleep (p<0.00) | CONSORT | | 12 | | 32.4 | | 3 | C | |
| Reza et al 2010 | RCT | 77 elderly residents with sleep problems | Acupressure at HT7, KI11, SP6 and Anmian | Researcher (trained for study) | Improvement compared to control for subjective sleep quality (p=0.028), sleep latency (p=0.001), sleep duration (p=0.007), habitual sleep efficiency (p=0.028) and sleep disturbance (p=0.013). | CONSORT | | 18 | | 48.6 | | 8 | B | |
| Sun et al 2009 | RCT | 44 elderly residents with sleep problems | Acupressure on HT7 compared to light touch | Researchers trained for study | Improved sleep (p<0.05) | CONSORT | | 30 | | 81.1 | | 12 | A | |
| Harris et al 2005 | Crossover | 39 students | Acupressure on LR4, ST36, KI1, BL10 compared to relaxation | Self | Greater alertness score (p=0.019). | CONSORT | | 22 | | 59.5 | | 14 | A | |
| Chan et al 2006 | Single group | 13 elderly residents with sleep problems | Behavioural group intervention including acupressure | TCM practitioner | Sleep improved (p<0.05) | TREND | | 19 | | 35.8 | | none | C | |
| Chen et al 1999 | Block experimental | 246 elderly residents with sleep problems | Acupressure on GV20, GB20, BL18, HT7 compared to sham | Researcher (trained in TCM) | Improved sleep (p<0.001) | CONSORT | | 22 | | 59.5 | | 14 | A | |
| **Mental health Category** **2** | | | | | | | | | | | | | | |
| **Anxiety/stress (3 studies)** | | | | | | | | | | | | | | |
| **Reference** | **Design** | **Total sample** | **Intervention** | **Administration** | **Results** | **Reporting** | | | | | | | **Grading** | |
|  |  |  |  |  |  | **Checklist** | | **score** | | **% score*** | | **Stricta score** |  | |
| Agarwal et al 2005 | RCT | 76 surgery patients | Acupressure extra 1 point compared to control | Researcher | Anxiety reduced (p<0.001), stress reduced (p<0.001) | CONSORT | | 16 | | 43.2 | | 8 | B | |
| Moriarty 2007 | single group | 25 pregnant women | Acupressure on LI4, GB34, ST36, SP6, KI3, LI3 and BL60 | researcher trained in acupressure | Reduced heart rate p= 0.003, anxiety (p=0.0001), tension (p=0.0001) and diastolic blood pressure (p=0.033) | TREND | | 29 | | 54.7 | | 14 | B | |
| Fassoulaki et al 2007 | within subjects | 12 healthy volunteers | Extra 1 acupressure compared to sham and control | n/s probably researcher | Bispectral index and verbal stress decreased (p=0.0001 and p=0.008) | TREND | | 25 | | 47.2 | | 10 | B | |
| **Dementia (2 studies)** | | | | | | | | | | | | | | |
| **Reference** | **Design** | **Total sample** | **Intervention** | **Administration** | **Results** | **Reporting** | | | | | | | **Grading** | |
|  |  |  |  |  |  | **Checklist** | | **score** | | **% score*** | | **Stricta score** |  | |
| Lin et al 2009 | RCT | 133 elderly residents | Acupressure on GB20, GV20, HT7, PC6, SP6 | staff with basic TCM training | Reduced agitation (p=0.001), aggression (p=0.001) and physically non aggressive behaviour (p=0.02) | CONSORT | | 16 | | 43.2 | | 13 | B | |
| Yang et al 2007 | within subjects | 31 elderly residents | Acupressure on GB20, GV20, HT7, PC6, SP6 | researcher | Improvement in all outcomes (p<0.001) | TREND | | 28 | | 52.8 | | 13 | C | |
| **Renal disease** (5 studies) **Category** **2** | | | | | | | | | | | | | | |
| **Reference** | **Design** | **Total sample** | **Intervention** | **Administration** | **Results** | **Reporting** | | | | | | | **Grading** | |
|  |  |  |  |  |  | **Checklist** | | **score** | | **% score*** | | **Stricta score** |  | |
| Cho and Tsay 2004 | RCT | 62 renal patients | Acupressure on ST36, SP6, KI3, KI1 compared to routine care | n/s | Reduced fatigue (p<0.001) and depression (p=0.03) | CONSORT | | 14 | | 37.8378 | | 15 | B | |
| Tsay and Chen 2003 | RCT | 98 renal patients | Acupresure on HT17 and KI1 compared to sham and control | researcher (trained) | Sleep improved compared to control (p<0.01) | CONSORT | | 13 | | 35.1351 | | 9 | B | |
| Tsay et al 2003 | RCT | 98 renal patients | Acupressure on HT17 and KI11 compared to sham and control | researcher (trained) | Sleep improved compared to control (p=0.003) | CONSORT | | 17 | | 45.9459 | | 14 | A | |
| Tsay 2004 | RCT | 106 renal patients | Acupressure on KI1, ST36, GB34 and SP6 compared to sham and control | researcher (trained) | Fatigue improved (p=0.02) compared to control | CONSORT | | 16 | | 43.2432 | | 12 | A | |
| Tsay et al 2004 | RCT | 106 renal patients | Acupressure on KI1, ST36, GB34 and SP6 compared to electrical stimulation and control | researcher (trained) | Fatigue, sleep and depression improved compared to control (p<0.001) | CONSORT | | 14 | | 37.8378 | | 12 | A | |
| **Respiratory (COPD, asthma etc)** (6 studies) | | | | | | | | | | | | | | |
| **Reference** | **Design** | **Total sample** | **Intervention** | **Administration** | **Results** | **Reporting** | | | | | | | **Grading** | |
|  |  |  |  |  |  | **Checklist** | | **score** | | **% score*** | | **Stricta score** |  | |
| Maa et al 2007 | RCT | 35 bronchiectasis outpatients | Acupressure on LU1, LU5, LU10, ST40 and ST36 compared to sham and control | self | Sputum reduced (p=0.03) and respiratory activity improved (p=0.01) | CONSORT | | 21 | | 56.8 | | 14 | B | |
| Maa et al 2003 | RCT | 41 COPD patients | Acupressure on LU1, GV14, Extra17, PC6, ST36 compared to standard care | self | Respiratory score improved (p=0.02) | CONSORT | | 18 | | 48.6 | | 12 | C | |
| Wu et al 2004 | RCT | 44 COPD patients | Acupressure on GV14, CV22, BL13, BL23, LU10 compared to sham points | researcher | Improved dyspnoea (p<0.05), fatigue (p<0.01) and activity (p<0.001 | CONSORT | | 18 | | 48.6 | | 12 | A | |
| Wu et al 2007 | Randomised, block experimental design | 44 COPD patients | Acupressure on GV14, CV22, BL13, BL23, LU10 compared to sham. | Researcher trained for study | Depression, dyspnea and oxygen saturation improved (p<0.001). | CONSORT | | 18 | | 48.6 | | 16 | A | |
| Maa et al 1997 | Crossover | 31 patients on pulmonary rehabilitation | Acupressure on LU1, LU2, LU10, LI4,GV14, PC8, ST36 compared to sham | self | Reduced dyspnoea (p=0.009) | CONSORT | | 22 | | 59.5 | | 6 | B | |
| Tsay et al 2005 | Blocking design trial | 52 COPD patients | Acupressure on LU14, PC6, HT7 compared to sham | Nurse TCM trained | Improved dyspnoea (P = 0.009), anxiety (P = 0.011) Heart rate (p=0.005) and respiratory rate (P < 0.0001) | CONSORT | | 19 | | 51.4 | | 11 | A | |
| **Measures of anaesthesia/consciousness (3 studies): Category 3** | | | | | | | | | | | | | | |
| **Reference** | **Design** | **Total sample** | **Intervention** | **Administration** | **Results** | **Reporting** | | | | | | | **Grading** | |
|  |  |  |  |  |  | **Checklist** | | **score** | | **% score*** | | **Stricta score** |  | |
| Litscher 2004 | RCT | 25 healthy volunteers | Acupressure on yintang compared to placebo | TCM practitioner | Bispectral index and spectral edge frequency reduced (p<0.001) | CONSORT | | 13 | | 35.1 | | 12 | C | |
| Fassoulaki et al 2003 | Crossover | 25 healthy volunteers | Acupressure on Extra 1 point compared to sham point | nurse | Bispectral index reduced (p<0.001) | TREND | | 26 | | 49.1 | | 10 | C | |
| Dullenkopf et al 2004 | Within subjects | 15 unsedated volunteers | Acupressure on Extra 1 compared to control point | researcher | Acupressure influenced Autogressive index and reduced stress levels | TREND | | 21 | | 39.6 | | 5 | C | |
| **Stroke (3 studies): Category 2** | | | | | | | | | | | | | | |
| **Reference** | **Design** | **Total sample** | **Intervention** | **Administration** | **Results** | **Reporting** | | | | | | | **Grading** | |
|  |  |  |  |  |  | **Checklist** | | **score** | | **% score*** | | **Stricta score** |  | |
| Kang et al 2009 | RCT | 56 stroke patients | Meridian acupressure compared to control | researcher | significant differences in functions of affected upper extremities (grip p=0.020, pain p=0.017, edema p=0.005, wrist flexion p = 0.002, wrist extension p < 0.001, elbow flexion p = 0.020, shoulder flexion p < 0.001, shoulder extension p < 0.001), activity of daily living (p<0.001) and depression (p=0.001) | CONSORT | | 13 | | 35.1 | | 6 | B | |
| McFadden and Hernandez 2010 | RCT (crossover) | 13 post-stroke volunteers from community | Individualised acupressure | Acupressure practitioner | Reduced heart rate (p=0.043) | CONSORT | | 22 | | 59.5 | | 13 | B | |
| Shin and Lee 2007 | RCT | 30 stroke patients with shoulder pain | Acupressure compared to aromatherapy acupressure LI15, SI9, TE14, GB21, SI11, SI12 | Not clear (probably researcher) | Reduced pain in acupressure group (p=0.001) | CONSORT | | 20 | | 54.1 | | 11 | B | |
| **Eyesight (2 studies)** | | | | | | | | | | | | | | |
| **Reference** | **Design** | **Total sample** | **Intervention** | **Administration** | **Results** | **Reporting** | | | | | | | **Grading** | |
|  |  |  |  |  |  | **Checklist** | | **score** | | **% score*** | | **Stricta score** |  | |
| Sun 2006 | RCT | 40 schoolchildren | Eye exercise including pressure on unspecified points (described as Yannei, Yanshang, Yanwai Yanxia), and GB20 | Researchers | Eyesight improved P<0.01 | CONSORT | | 9 | | 24.3 | | 9 | C | |
| Yeh et al 2007 | Controlled nonrandomised trial | 70 schoolchildren | Acupressure on BL2, BL1, ST1 ST2, Extra2, GV20, GB20, LI4 | n/s | Improved visual health knowledge, visual acuity, and refractive error | CONSORT | | 13 | | 35.1 | | 12 | B | |
| **Weight (loss/gain) (2 studies)** | | | | | | | | | | | | | | |
| **Reference** | **Design** | **Total sample** | **Intervention** | **Administration** | **Results** | **Reporting** | | | | | | | **Grading** | |
|  |  |  |  |  |  | **Checklist** | | **score** | | **% score*** | | **Stricta score** |  | |
| Chen et al 2007 | RCT | 40 premature babies | Acupressure on ST36, CV12, KI1 and meridian massage of spleen and stomach meridian | Nurse TCM trained | Babies gained weight in experimental group (p=0.038) | CONSORT | | 18 | | 48.6 | | 14 | B | |
| Elder et al 2007 | RCT | 90 overweight community members | Tapas Acupressure Technique (holding a pose which applies pressure to GB21, BL1 and yin tang) | self | Maintained weight loss compared to self directed study (p=0.09) and qi gong (p=0.00) | CONSORT | | 21 | | 56.8 | | 10 | A | |
| **Other (Inconclusive)** | | | | | | | | | | | | | | |
| **Reference** | **Design** | **Total sample** | **Intervention** | **Administration** | **Results** | **Reporting** | | | | | | | **Grading** | |
|  |  |  |  |  |  | **Checklist** | | **score** | | **% score*** | | **Stricta score** |  | |
| **Cancer side effects (other than N&V)** | | | | | | | | | | | | | | |
| Chao et al 2009 | SR | 26 studies | Acupoint stimulation for breast cancer AEs | N/A | Acustimulation is useful for breast cancer N&V | CASP | | 9 | | 90 | | N/A | A | |
| **Cardiovascular (3 studies)** | | | | | | | | | | | | | | |
| Ballegaard et al 2004 | Nonrandomised | 169 patients with angina | Acupressure on CV17, BL14 and 15 as part of integrated program | self | Accumulated risk improved over time (p<0.05) | TREND | | 35 | | 66.1 | | N/A | | B~~A~~ |
| Ballegaard et al 1999 | Open prospective study | 105 patients with angina | Acupressure on CV17, BL14, BL15 | Self | Cost saving over 5 years of $32,000 per patients | N/A | | | |  | | 11 | | ungraded |
| Li et al 2007 | Controlled trial | 30 patients with peripheral arterial occlusive disease | Acupressure on GB34, ST36, SP9, SP6 compared to control | n/s | Decreased blood flow (p>0.05). | CONSORT | | 8 | | 21.6 | | 10 | | C |
| **Chronic Fatigue Syndrome (1 study)** | | | | | | | | | | | | | | |
| Yao et al 2007 | One group | 85 patients with CFS | Acupressure massage | Researcher | Treatment was effective in 91.8% of cases | TREND | | 15 | | 28.3 | | 7 | | ungraded |
| **Diabetes (1 study)** | | | | | | | | | | | | | | |
| Jin et al 2009 | RCT | 80 patients with diabetes | Whole body acupressure | Acupressure practitioners | Hyperlipidemia, hypertrophy and kidney function improved (p<0.05) | CONSORT | | 19 | | 51.4 | | 14 | | A |
| **Gagging (1 study)** | | | | | | | | | | | | | | |
| Lu et al 2000 | RCT | 109 dental patients | Acupressure at PC6 compared to sham | n/s (probably conventional practitioner) | Reduced gagging (p=0.001) | CONSORT | | 7 | | 18.9 | | 13 | | B |
| **Gastrointestinal motility (2 studies)** | | | | | | | | | | | | | | |
| Chen et al 2006 | RCT | 64 neurological patients | LI4, CV12, ST25 |  | Improved bowel movement (p<0.05) | CONSORT | | 14 | | 37.8 | | 13 | | C |
| Chen et al 2003 | RCT | 41 hysterectomy patients | PC6, ST36, SP6 | Nurse (trained in acupressure) + self | Increased GI motility (p<0.05) | CONSORT | | 11 | | 29.7 | | 11 | | B |
| **General (‘nursing practice’) (1 review)** | | | | | | | | | | | | | | |
| Ma et al 2007 | SR | 71 studies |  | N/A | 97.2% of articles had positive effects | CASP | | 1 | | 10 | | N/A | | C |
| **Mechanism (1 study)** | | | | | | | | | | | | | | |
| Sugiura et al 2007 | Uncontrolled | 22 students | Acupressure on KI1 and Japanese points souk-shin, shitsu-min | Nurse | Heart rate decreased (p<0.05) | N/A | | | | | | 12 | | ungraded |
| **Nocturnal enuresis (1 study)** | | | | | | | | | | | | | | |
| Yuksek et al 2003 | RCT | 24 patients with nocturnal enuresis | Acupressure at GV4, GV15, GV20, BL23, BL28, BL32, HT7, HT9, ST36, SP4, SP6, SP12, CV2,CV3,CV6, KI3 and KI5. | Parent | No significant differences | CONSORT | | 8 | | 21.6 | | 4 | | C |
| **Sexual dysfunction (1 study)** | | | | | | | | | | | | | | |
| Ventegodt et al 2006 | Uncontrolled | 20 patients with sexual problems | Vaginal acupressure (individualised) | Acupressure practitioner | Improvements in symptoms (p<0.05), physical health (p=0.042), mental health (p=0.012), sexual ability (p=0.003) and quality of life (p=0.003 and 0.007) | TREND | | 16 | | 30.9 | | 5 | | C |
| n/s: not specified | | | | | | | | | | | | | | |

1. Grading based on rigour of the study, STRICTA score, quality of reporting and study design

   A (good): Least bias and results are valid; a primary study that uses a high quality study design and adheres to commonly held concepts of high quality

   B (fair/moderate): Susceptible to some bias, but not sufficient to invalidate the results; a primary study that does not meet all the criteria in category A.

   C (poor): Significant biases that may invalidate the results; a primary study with serious errors in design, analysis or reporting.

   *% items reported out of possible items on checklist [↑](#footnote-ref-2)
2. Grading based on rigour of the study, STRICTA score, quality of reporting and study design

   A (good): Least bias and results are valid; a primary study that uses a high quality study design and adheres to commonly held concepts of high quality

   B (fair/moderate): Susceptible to some bias, but not sufficient to invalidate the results; a primary study that does not meet all the criteria in category A.

   C (poor): Significant biases that may invalidate the results; a primary study with serious errors in design, analysis or reporting. [↑](#footnote-ref-3)
